# Gene sequencing and result analysis of balanced translocation carriers by third-generation gene sequencing technology

**DOI:** 10.1038/s41598-022-20356-8

**Published:** 2023-04-28

**Authors:** Xiaoqi Zeng, Dandan Lin, Danhong Liang, Jingwen Huang, Jinsong Yi, Dianliang Lin, Zhengmian Zhang

**Affiliations:** 1 Fujian Provincial Sperm Bank, Fujian Maternity and Child Health Hospital College of Clinical Medicine for Obstetrics and Gynecology and Pediatrics, Fujian Medical University, Fuzhou, China; 2 Obstetrics Department of Longyan First Hospital of Fujian Medical University, Fuzhou, China

**Keywords:** Epigenetics, Sequencing

## Abstract

Because the total gene copy number remains constant and all genes are normally expressed, carriers of balanced chromosomal translocations usually have a normal phenotype but are able to produce many different types of gametes during meiosis, and unbalanced gametes lead to increased risks of infertility, recurrent spontaneous abortion, stillbirth, neonatal death or malformations and intellectual abnormalities in offspring. The key to balanced translocations lies in finding the breakpoints, but current genetic testing techniques are all short-read sequencing, with the disadvantage of procedural complexity and imprecision for precisely identifying the breakpoints. The latest third-generation sequencing technology overcomes these drawbacks and uses robust long-read sequencing to accurately and rapidly detect genome-wide information and identify breakpoint locations. In this paper, we performed whole genome long-read sequencing using an Oxford Nanopore sequencer to detect the breakpoints of 4 balanced chromosomal translocation carriers. The results showed that employing about ~ 10× coverage confirmed 6 of the 8 breakpoints, of which, 2 had microdeletions/insertions identified near the breakpoints and 4 had breakpoints that disrupted the normal gene structure and were simultaneously tested for genome-wide structural variation (SV). The results show that whole genome long-read sequencing is an efficient method for pinpointing translocation breakpoints and providing genome-wide information, which is essential for medical genetics and preimplantation genetic testing.

## Introduction

Structural variation (SV) is usually defined as a region of DNA that changes in copy number (deletions, insertions, and duplications), orientation (inversions), or chromosomal position (translocations) between individuals. SV can be balanced, with no loss or gain of genetic material, such as inversions of genetic segments or translocations of intra- or interchromosomal DNA segments, or unbalanced, in which a portion of the genome is lost or duplicated , called copy number variation (CNV)^1^. Chromosomally balanced translocation carriers usually have a normal phenotype because the total gene copy number remains constant and all genes are normally expressed. However, in a few cases, translocations have been reported to be associated with various diseases^2–4^. It can produce at least 18 different types of gametes during meiosis, of which only one type is normal, one type is balanced, and the rest carry unbalanced chromosomal changes, which are derivatives of terminal sequence duplications and deletions on either side of the breakpoint^5,6^. Fertilization of unbalanced gametes may lead to infertility, recurrent spontaneous abortions, stillbirths, neonatal death or malformations and intellectual abnormalities in offspring. Fertilization reproduction of normal/balanced gametes depends on the chromosome involved, breakpoint location, segregation pattern, and sex of the translocation carrier^7,8^, with approximately 1/500 to 1/1000 live births being balanced translocation carriers^9^. The ability to identify fully normal embryos through the precise localization of balanced translocation breakpoints may prevent future generations from facing the same reproductive dilemma. Breakpoints are a common phenomenon that occurs during chromosomal recombination during meiosis and can lead to genetic variation, and patients with pathogenic translocations usually have breakpoints within or near disease genes^10^.

In some special balanced translocation carriers such as: when the reciprocal translocation involves the X chromosome, the phenotype of balanced carriers is unpredictable due to random inactivation of the X chromosome during early embryonic development^11^, at which point the best strategy is to select only euploid non carrying balanced translocated embryos. Even more so, the difficulty of obtaining normal/balanced embryos is increased by the presence of balanced translocations in both partners, which have been reported in several cases of consanguineous marriages in which offspring underwent pregnancy termination due to different phenotypic abnormalities^12–15^. Preimplantation genetic diagnosis (PGD) enables embryo biopsy of chromosomal translocation carriers to select normal or balanced euploid embryos for intrauterine transfer, which will effectively reduce miscarriage and improve live birth rates^16^.

Various methods are currently available for breakpoint identification, such as single nucleotide polymorphism (SNP) array, microdissection, next-generation sequencing, allele mapping identification, paired end sequencing by haplotype linkage analysis (PGH)^15,17–20^. However, translocation breakpoints are often associated with highly repetitive sequences, and determining precise breakpoints in highly repetitive and variable translocation regions remains unstable and inaccurate^21^. At present, the inadequate sequencing accuracy and short fragment length are still the shortcomings of short-read sequencing that cannot be ignored. Third generation sequencing addresses these issues, and the long read lengths (> 10 kb on average) it produces will greatly improve SV detection, finding translocation breakpoints regardless of whether the SV is located in a repetitive region and can cover up to two million bases up—and downstream of the breakpoint. Two technologies are currently available: single molecule real-time sequencing (SMRT) by Pacific Biosciences and Oxford Nanopore sequencing (ONT) by Oxford Nanopore technologies.Both allow sequencing of non amplified native DNA with ultra long linear read lengths (1–100 kb/s) and rapid sequencing times (2–10 h)^22,23^. The use of third-generation sequencing for structural chromosomal rearrangements, gene fusions and deletions or insertions involving DNA bases has been described, with fewer reports for preimplantation genetic testing (PGT) applications^24–26^. In this study, we resequenced couples with reciprocal balanced translocations of known chromosomal status who were either carriers of chromosomal balanced translocations or patients with recurrent implantation failure, explored the use of third-generation sequencing for higher sequencing depth, analysis of longer genomic fragments, and further analysis of structural rearrangement details in couples with balanced translocations to provide more accurate genetic information for PGT.

## Materials and methods

### Samples

Case 1: a young couple, wife 29 years, husband 30 years, with 5 years of infertility. The wife's karyotype analysis prompted: 46, XX, t(X;3)(p22.3;q26.2); The husband's semen was examined for Oligozoospermia (concentration 5.9× 10^6^/ml, total motility 7.9%, normal morphology 3%), suggested by karyotype analysis: 46, XY, t(13;16)(q34;q12.1). There was no consanguinity in either side. Assisted reproduction assisted pregnancy was performed with PGD by array comparative genomic hybridization (aCGH) technique and transfer of a balanced/normal D5 frozen embryo prompted by amniocentesis at 19 + weeks: 46, XN, t(13;16)(q34;q12.1) pat.

Case 2: The wife is 30 years old and the husband is 33 years old with 3 years of infertility. The wife's karyotype analysis prompted: 46, XX, t(1;3)(p22;p13); The husband's karyotype was normal: 46, XY. All four cycles of assisted reproduction failed to achieve pregnancy, none of the three cycles of artificial insemination with husband's sperm (AIH) were implanted, and one cycle of in vitro fertilization (IVF) cancelled PGD due to no good quality blastocysts.

Case 3: The wife is 26 years old and the husband is 26 years old, with 3 years of infertility. The wife's karyotype was normal: 46, XX; the husband's karyotype analysis was suggestive: 46, XY, t(2; 4)(q23; q32). Two cycles of IVF assisted pregnancies failed, four good quality blastocysts were obtained in the first cycle, one D5 balanced/normal embryo was obtained by PGD biopsy, one D6 good quality blastocyst was obtained by ET and one euploid unbalanced embryo was retrieved by MaReCs PGD.

### Sample collection

Blood samples were taken to freshly extract DNA, which was forbidden from freezing. High quality genomic DNA was isolated from each sample using the SDS method. DNA quality and concentration were tested by 0.75% agarose gel electrophoresis, nanodrop one spectrophotometer (Thermo Fisher Scientific) and qubit 3.0 fluorometer (life technologies, Carlsbad, CA, USA).

### Library preparation and sequencing

The ONT ligation sequencing protocol LSK109 was chosen to construct the sequencing library and the prepared library was loaded onto the R9 chip for 1D sequencing, the library preparation process took 1h30min. The following is the procedure: Genomic DNA was sheared into fragments of approximately 5–25 kilobases using meagruptor 2 (Diagenode, B06010002) followed by size selection (10–30 kilobases) with the bluepippin apparatus (Sage Science, MA) to remove small DNA fragments. Sheared deoxyribonucleic acid was end repaired using the NEBNext Ultra II End-Repair/dA-tailing Module (NEBnext ultra II end repair kit, New England Biolabs, USA)and then end repaired with (1×) Ampure beads (Beckmann Coulter, USA)for purification. This module repairs fragmented DNA to produce DNA with 5′ phosphorylation and 3 ′ ends. The end repaired DNA was incubated with NEB blunt/TA mix (New England Biolabs, MA, USA) using (0.6×) Ampure beads (Beckmann Coulter, USA) elution, repaired DNA was used for adapter ligation, purified products were sequenced adapter ligation using the SQKLSK109 ligation kit, cleaned end repaired DNA was quantified using the Qubit dsDNA assay kit, and purified libraries were loaded onto starting R9.4 Spot-On flow cells and sequenced using a PromethION sequencer (Oxford Nanopore technologies, Oxford, UK) running for 48 h at Wuhan benagen tech solutions company limited, Wuhan, China.

### Data analysis

The raw data were subjected to base calling analysis using Oxford Nanopore GUPPY software (v0.3.0). Raw sequencing data were in FAST5 format and first converted to FASTQ format using the MINKNOW base caller. Structural variants were then called using a workflow combining Minimap2 and NanoVar, an optimized structural variant caller that utilizes the low depth (~ 10× coverage) generated by Oxford Nanopore Technology whole genome sequencing data. Briefly, long reads were aligned to the human reference genome (GRCh37) using minimap2 (version 2.17-r941) with "—ax map ont – MD " parameters, followed by SV calling performed by NanoVar with default parameters. To maximize the sensitivity of translocation discovery, all split reads mapping to different chromosomes were collected and their detailed mapping information was recorded using custom Perl scripts. As a quality check, the integrative genomics viewer (IGV) was used to manually inspect the translocations detected in the targeted regions. Removal of sequences with average mass value less than or equal to 7.

### Ethics statement

The studies involving human participants were reviewed and approved by ethics committee of Fujian Provincial Maternal and Child Health Hospital. All couples received detailed genetic counseling before receiving PGD, and were informed of the risks associated with reciprocal translocation carriers, the advantages and disadvantages associated with the PGD process, and the advantages and disadvantages of third-generation sequencing technology. The patients/participants provided their written informed consent to participate in this study. The research, including human subjects, human data and material, has been performed in accordance with the Declaration of Helsinki.

## Results

**Figure 1:**
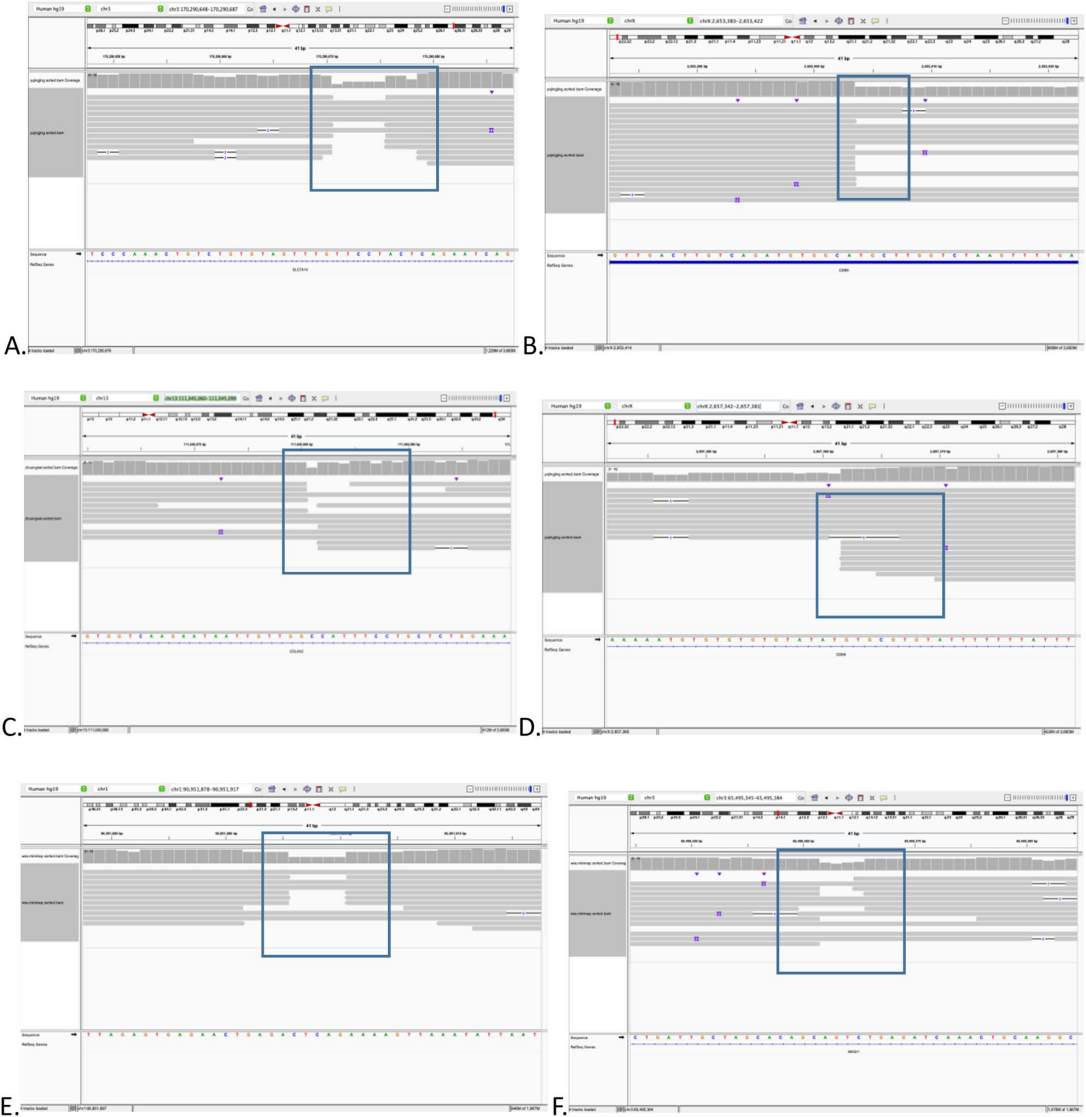
Long read sequencing of translocation breakpoints in samples 1–2 and the base sequences around the breakpoints are shown in the IGV, the area in the red box is the breakpoint reads. Colored capital letters at the bottom of the IGV represent base information. Reads breakpoint location display in IGV: (**A**) chr3: 170290668–170290678. (**B**) chrX:2653403–2657362. (**C**) chr13: 111045080–111045082. (**D**) chr16: 46392636–46398439/46404543. (**E**) chr1: 90951892–90951904. (**F**) chr3: 65495358–65495367.

We found six breakpoints in three samples in cases 1 and 2. The locations of the breakpoints were consistent with the karyotyping results.The balanced translocation carrier sequencing results DNA fragments were compared to the reference human genome (*GRCh37/hg19*) and breakpoint positions are indicated in IGV (as in Fig. 1), while in case 3 the translocation carrier 46, XY, t(2;4)(q23;q32) sequencing results were compared to the reference human genome (*GRCh37/hg19*) no breakpoint sequence information was obtained.

The 46, XX, t(X;3)(p22.3; q26.2) translocation carrier breakpoint in case 1 has 8 reads in IGV locating the position at chr3:170290668–170290678 and chrX:2653403–2657362, whose detection results show a 4 kb deletion at the breakpoint. 8 reads in the 46, XY, t(13;16)(q34; q12.1) translocation carrier breakpoint localized the position to chr13: 111045080–111045082 and chr16: 46392636–46398439/46404543, whose detection results showed a 6/12 kb deletion at the breakpoint. The 46, XX, t(1; 3)(p22; p13) translocation carrier breakpoint in case 2 had 7 reads mapping in IGV to chr1:90951892–90951904 and chr3:65495358–65495367.

**Figure 2:**
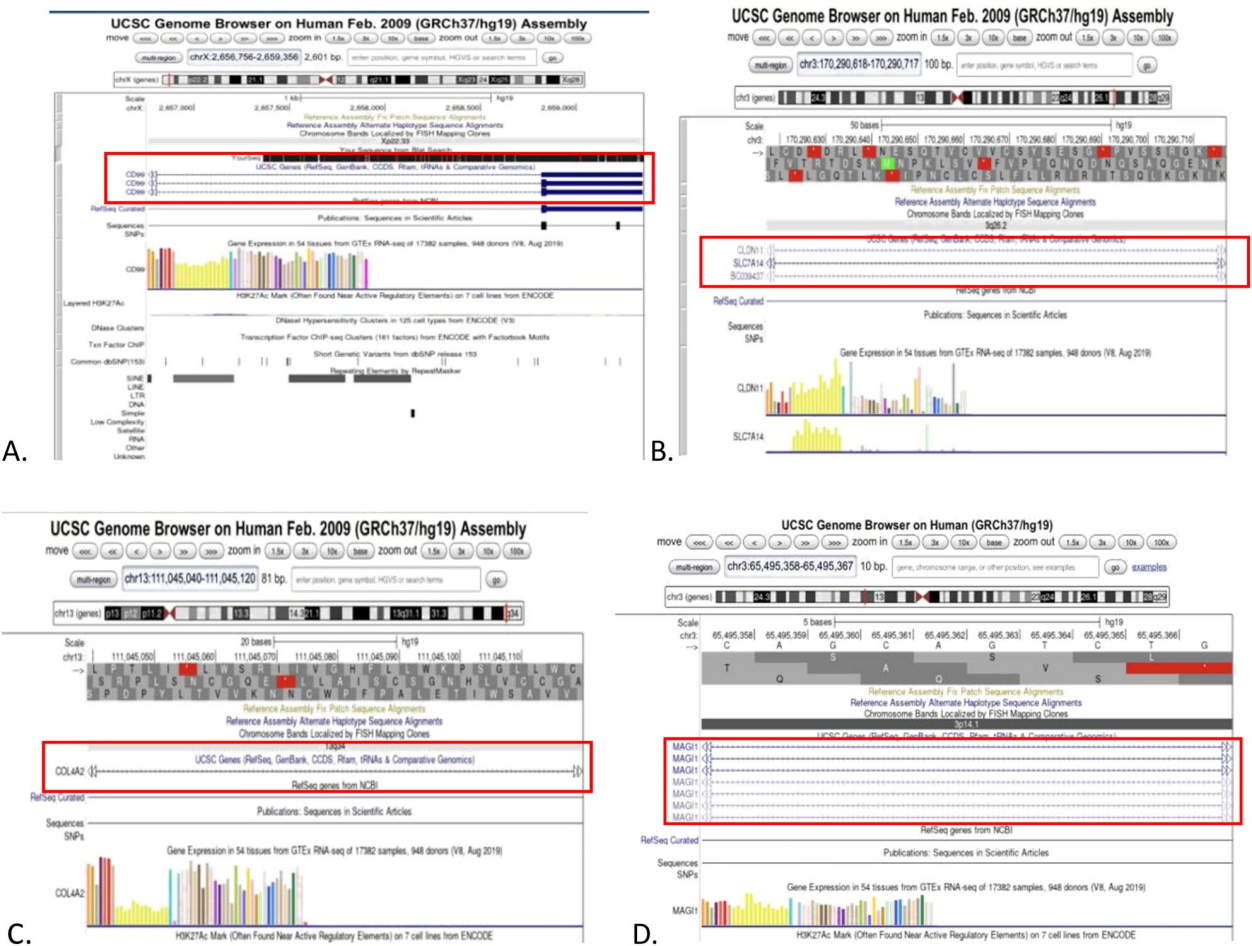
The balanced translocation carrier sequencing results DNA fragments were compared to the reference human genome (*GRCh37/hg19*): (**A**) chrX:2653403–2657362 (**B**) chr3:170290668–170290678 (**C**) chr13: 111045080–111045082 (**D**) chr3: 65495358–65495367.

Inspection of these breakpoints in the UCSC Genome Browser (Table 1; Fig. 2) revealed disruption of the *COL4A2* gene by the translocation carrier 46, XY, t(13; 16)(q34; q12.1) chromosome 13 breakpoint in case 1, which encodes a protein important for angiogenesis and tumor growth suppressors, and the chromosome 16 breakpoint does not involve a functional gene. Translocation carriers with a 46, XX, t(X; 3)(p22.3; q26.2) chromosome 3 breakpoint disrupting the *CLDN11*, *SLC7A14*, *BC039437* genes encoding proteins that are important components of the central nervous system.*SLC7A14* associated with recessive disease, and *BCO039437* that function similarly to *CLDN11* encoded proteins. The X chromosome breakpoint disrupts the *CD99* gene structure, which is associated with a variety of malignant neoplastic diseases, and the gene breakpoint occurs in an *AluSc* element. The chromosome 3 breakpoint in the translocation carrier 46, XX, t(1; 3)(p22; p13) in case 2 disrupts the *MAG11* gene, but no pathogenicity has been reported for this gene so far and no functional gene has been implicated in chromosome 1. These breakpoints disrupt the gene structure, leading to the exchange of chromosomal segments, as a portion of the gene on one chromosome is transferred to the other chromosome, thereby impairing gene function. However, with the exception of primary infertility, there was no apparent effect on the phenotype of the carriers from whom the sample was obtained.

**Table 1:** Table of genes and functions disrupted by breakpoints.

Breakpoint location	Gene	Coding functions
chr3: 170290668–170290678	*CLDN11*	Involvement in central nervous system composition
	*SLC7A14*	Associated with recessive disorders
	*BC039437*	Involvement in central nervous system composition
chr13: 111045080–111045082	*COL4A2*	Angiogenesis and tumor growth inhibitors
chrX: 2653403–2657362	*CD99*	Multiple malignant neoplastic diseases
chr3: 65495358–65495367	*MAG11*	Pathogenicity has not been reported

Diagram of breakpoints aligned to the reference genome in UCSC(the area in the red box is the breakpoint disruption gene): A.chrX: *CD99* B.chr3: *CLDN11*, *SLC7A14*, *BC039437* C.chr13: *COL4A2* D.chr3: *MAG11.*

### SV detection

**Figure 3:**
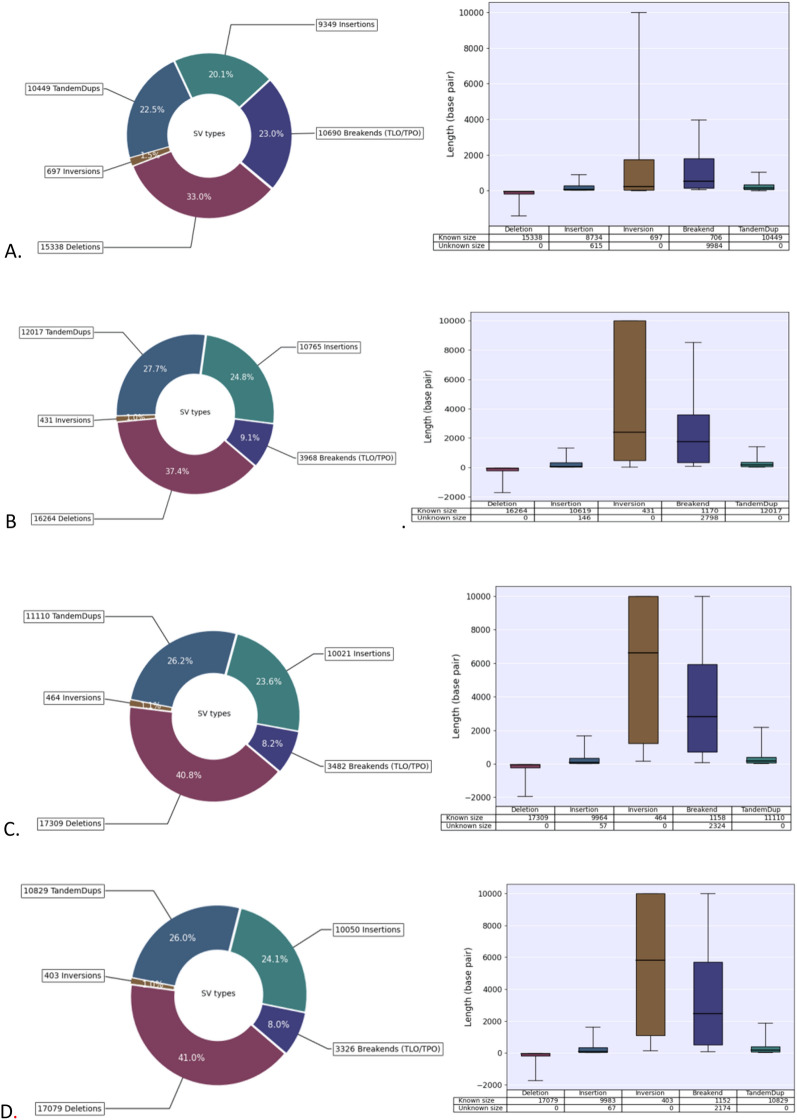
The pie chart is the SV percentage, and the bar chart is the SV base length. Results of whole gene SV testing: (**A**) 46, XX, t(X;3)(p22.3;q26.2) (**B**) 46, XY, t(13;16)(q34;q12.1). (**C**) 46, XX, t(1;3)(p22;p13) (**D**) 46, XY, t(2;4)(q23;q32).

Whole genome testing was performed on the samples, and the results of SV testing are shown in Fig. 3. In case 1, the translocation carrier 46, XX, t(X;3)(p22.3; q26.2) detected a total of 10,449 kb of tandem repeats, 697 kb of inversions, 15,338 kb of deletions, and 9349 kb of insertions. Translocation carriers 46, XY, t(13; 16)(q34; q12.1) detected tandem duplications totaling 12,017 kb, inversions totaling 431 kb, and deletions totaling 16264 kb. Translocation carriers 46, XX, t(1; 3)(p22; p13) in case 2 detected a total of 11110 kb in tandem duplications, 464 kb in inversions, 17309 kb in deletions, and 10021 kb in insertions. Carrier 46, XY, t(2; 4)(q23; q32) in case 3 detected a total of 10829 kb of tandem duplications, 403 kb in inversions, 17,079 kb in deletions and 10,050 kb in insertions.

## Discussion

Currently, karyotyping is the most widely used technique for clinical diagnosis of chromosomal translocations, however, it is a low resolution method that can only find the breakpoints at the chromosomal level and cannot determine the exact breakpoints. FISH is limited to the detection of very few chromosomes and since it relies on fluorescent labelling, the results are sometimes inconclusive due to ambiguous optical signals and complex sample preparation procedures^27^. NGS technology enabled high-resolution and high-throughput analysis with comprehensive chromosome screening (CCS) of all 24 chromosomes by using aCGH or next-generation sequencing (NGS). Multiple clinical trials have shown that the use of CCS increases pregnancy and live birth rates per transfer cycle^28,29^. But NGS creates difficulties due to defects in their read lengths (35–600 bp) when breakpoints are located in complex repeat regions with low mapping rates, making it difficult to accurately detect their location. In identifying translocation breakpoints by long-range pairing, Hu et al. used laser microdissection to identify SNP markers associated with the translocation associated allele^10^. It is a NGS based CCS approach to address the translocation carrier status of embryos, and while this approach can identify translocation affected alleles, it requires highly specialized equipment and complex sample preparation procedures, making it unlikely to be available for routine clinical use^30^. A SNP array based linkage analysis approach was also used to identify translocation free embryos. However, it has limited accuracy in breakpoint identification and linkage analysis^31^. MALBAC-NGS can increase the accuracy of breakpoint determination to 200 kbp, reducing the current range limit for informative SNPs to 1Mbp on either side of the breakpoint. MaReCs are a reliable method to distinguish the translocation carrier status of embryos from balanced translocation carriers. It can help a proportion of balanced translocation carriers select completely normal embryos, while reducing the transfer of embryos carrying balanced translocations, with the limitation that it requires the availability of reference embryos^21^.

In this paper we successfully identified and sequenced each breakpoint in three cases of balanced translocation carriers by long-read sequencing, and all six breakpoints were consistent with their corresponding karyotype results. Many previously unidentified minor structural variants were found at two breakpoints (translocation chromosomes 8 and 16). These results suggest that certain copy number deletions are often detected near the breakpoints. Microdeletions/insertions often occur in conjunction with translocations, and mutual chromosome translocations are usually accompanied by some additional rearrangements, such as deletions and duplications, which can involve a few base pairs or up to millions of bases^32^. Nanopore sequencing is a single-molecule long read sequencing technology that overcomes the limitations of short read sequencing, such as the inability to reliably resolve repetitive sequences or large genomic rearrangements, typified by long reads (> 1 kb to 2 Mb) and real-time sequencing. Compared with other platforms, Nanopore sequencing has many potential advantages, including the possibility of label free sequence determination of native DNA and RNA molecules without amplification and the ability to generate extremely long read lengths, with some groups reporting read lengths > 2 Mb^18^. This greatly increases the chance of obtaining chimeric reads overlapping breakpoint junctions.

Genomic structural variants generally refer to large length sequence changes and positional relationships across the genome and are rich in variant types, including long segmental sequence insertions or deletions (big indel), tandem duplications, chromosomal inversions, chromosomal translocations, copy number variations (CNV), and more complex forms of mosaicism. Compared to SNPs (single nucleotide polymorphisms), SVs make up a larger proportion of the variant base, have a greater impact on the genome, and, once altered, tend to have a major impact on the body of life. In humans, such structural variants have been associated with many diseases (including autism, obesity, schizophrenia, and cancer, among others); On plants, SVs are associated with many phenotypic variations and thus become increasingly important research areas. Most high-throughput sequencing technologies basically sequence short pieces of DNA (generally 150–300 bp), which is more difficult and less accurate in analyzing larger structural variations^1^. This analysis allowed us to perform statistics on SVs in our samples by contrasting them with reference genes, and since our study focused on translocations already identified by karyotype, we did not perform a more detailed analysis of SVs. But these data suggest that, on SV statistics, the adoption of long-read Nanopore sequencing is more likely to encompass entire structural variants and/or repetitive regions, leading to more accurate and precise structural variants and an improved understanding of structural variation and the role of structural variation in disease, evolution and genetic diversity.

The major drawbacks of the third-generation sequencing platform are the lower throughput and higher error rate, there was one failure case in this paper, the breakpoint position information was not detected, which would lead to two impacts due to the high cost and single base error rate of third generation sequencing, first, the cost of sequencing limits the sequencing depth, and the average sequencing of whole genome is 10× coverage.But not completely average, and some will be above 10× coverage, some locations below 10× coverage even less well covered. It is possible that the sequencing depth at the breakpoints of balanced translocations is too low to result in difficult detection; The second is the high Nanopore single base error rate, which, if at a relatively low sequencing depth, may lead to difficulties in information analysis, i. e. increasing the difficulty of aligning sequencing reads back to the reference genome. It is possible that there was genomic information at the breakpoints measured at the time of sequencing, but this was not detected due to alignment errors resulting from sequencing errors. But with the release of the platform and advances in flow cell design and sequencing chemistry, error rates as low as 3% can now be achieved^33^. For samples that test negative if testing is to be continued to find the break points can increase the Nanopore sequencing depth, as indicated by the 10× coverage was increased to 20× or 30× coverage.Greatly improve the probability of breakpoint detection. But the detection will cost quite a bit more. The site of the balanced translocation is mapped with high accuracy by mate-pair, this particular next-generation sequencing can generally be mapped to the 1–10 k interval, and then the mapped interval is examined by high depth next-generation sequencing or direct Sanger sequencing to find the precise break site.

Third generation sequencing holds great promise. The main applications of Pacbio sequencing include WGS, targeted sequencing, full-length mRNA sequencing, sequencing of complex populations, and detection of epigenetic modifications^32^. In a study reported by Chun et al. using Nanopore sequencing in a newly diagnosed AML patient, conventional karyotyping showed translocation t(10;12)(q22;p13) but RNA NGS detected *NUP98-NSD1* fusion transcripts from a known cryptic translocation t(5;11)(q35;p15). Rapid PCR-free Nanopore whole-genome sequencing yielded a 26,194 bp sequencing read and revealed the t(10;12) breakpoint to be *DUSP13* and *GRIN2B* in head-to-head configuration. This translocation was then classified as a passenger structural variant. The sequencing also yielded a 20,709 bp sequencing read and revealed the t(5;11) breakpoint of the driver *NUP98-NSD1* fusion. The identified DNA breakpoints also served as markers for molecular monitoring, in addition to fusion transcript expression by digital PCR and sequence mutations by NGS. This suggests that third-generation Nanopore sequencing is a simple and cost-effective workflow for DNA translocation detection^34^.

## Conclusion

Third generation sequencing technology can directly obtain genomic sequence information and simultaneously perform SV detection by performing long read sequencing, and has significant advantages in detecting gene minor variations, and can accurately identify translocation breakpoint position information for carriers of chromosomal balanced translocations, making genomic information more refined and extensive, which can provide accurate and rich genetic information for PGT. However, the technique is still more expensive to analyze, and the occurrence of experimental failure still needs further increased sample in-depth study.

## Data Availability

The raw required to reproduce these findings cannot be shared at this time as the data also forms part of an ongoing study.

## References

[1] Escaramís G, Docampo E, Rabionet R (2015). A decade of structural variants: Description, history and methods to detect structural variation. Brief Funct. Genomics..

[2] Utami KH, Hillmer AM, Aksoy I, Chew EG, Teo AS, Zhang Z, Lee CW, Chen PJ, Seng CC, Ariyaratne PN, Rouam SL, Soo LS, Yousoof S, Prokudin I, Peters G, Collins F, Wilson M, Kakakios A, Haddad G, Menuet A, Perche O, Tay SK, Sung KW, Ruan X, Ruan Y, Liu ET, Briault S, Jamieson RV, Davila S, Cacheux V (2014). Detection of chromosomal breakpoints in patients with developmental delay and speech disorders. PLoS ONE.

[3] Mikelsaar R, Nelis M, Kurg A, Zilina O, Korrovits P, Rätsep R, Väli M (2012). Balanced reciprocal translocation t(5;13)(q33;q12) and 9q31.1 microduplication in a man suffering from infertility and pollinosis. J. Appl. Genet..

[4] Sandberg AA, Meloni-Ehrig AM (2010). Cytogenetics and genetics of human cancer: Methods and accomplishments. Cancer Genet. Cytogenet..

[5] Carinci F, Pezzetti F, Scapoli L, Martinelli M, Avantaggiato A, Carinci P, Padula E, Baciliero U, Gombos F, Laino G, Rullo R, Cenzi R, Carls F, Tognon M (2003). Recent developments in orofacial cleft genetics. J. Craniofac. Surg..

[6] Munné S (2005). Analysis of chromosome segregation during preimplantation genetic diagnosis in both male and female translocation heterozygotes. Cytogenet. Genome Res..

[7] Ye Y, Qian Y, Xu C, Jin F (2012). Meiotic segregation analysis of embryos from reciprocal translocation carriers in PGD cycles. Reprod. Biomed. Online.

[8] Lledó B, Ortiz JA, Morales R, Ten J, de la Fuente PE, García-Ochoa C, Bernabeu R (2010). The paternal effect of chromosome translocation carriers observed from meiotic segregation in embryos. Hum. Reprod..

[9] Zhang L, Wei D, Zhu Y, Jiang W, Xia M, Li J, Yan J, Chen ZJ (2019). Interaction of acrocentric chromosome involved in translocation and sex of the carrier influences the proportion of alternate segregation in autosomal reciprocal translocations. Hum. Reprod..

[10] Fischer J, Colls P, Escudero T, Munné S (2010). Preimplantation genetic diagnosis (PGD) improves pregnancy outcome for translocation carriers with a history of recurrent losses. Fertil. Steril..

[11] Chen W, Ullmann R, Langnick C, Menzel C, Wotschofsky Z, Hu H, Döring A, Hu Y, Kang H, Tzschach A, Hoeltzenbein M, Neitzel H, Markus S, Wiedersberg E, Kistner G, van Ravenswaaij-Arts CM, Kleefstra T, Kalscheuer VM, Ropers HH (2010). Breakpoint analysis of balanced chromosome rearrangements by next-generation paired-end sequencing. Eur. J. Hum. Genet..

[12] Waters JJ, Campbell PL, Crocker AJ, Campbell CM (2001). Phenotypic effects of balanced X-autosome translocations in females: A retrospective survey of 104 cases reported from UK laboratories. Hum. Genet..

[13] Martinet D, Vial Y, Thonney F, Beckmann JS, Meagher-Villemure K, Unger S (2006). Fetus with two identical reciprocal translocations: Description of a rare complication of consanguinity. Am. J. Med. Genet. A.

[14] Tsuji K, Narahara K, Yokoyama Y, Ninomiya S, Yonesawa S, Hiramatsu Y, Masaoka H, Kudo N, Seino Y (1993). Reproductive risk in mating between two translocation carriers: Case report and review of the literature. Am. J. Med. Genet..

[15] Ozkul Y, Dundar M (2002). A family with two different chromosomal translocations. Ann. Genet..

[16] Cheng D, Yuan S, Hu L, Yi D, Luo K, Gong F, Lu C, Lu G, Lin G, Tan YQ (2021). The genetic cause of intellectual deficiency and/or congenital malformations in two parental reciprocal translocation carriers and implications for assisted reproduction. J. Assist. Reprod. Genet..

[17] Coonen E, Martini E, Dumoulin JC, Hollanders-Crombach HT, de Die-Smulders C, Geraedts JP, Hopman AH, Evers JL (2000). Preimplantation genetic diagnosis of a reciprocal translocation t(3;11)(q27.3;q24.3) in siblings. Mol. Hum. Reprod..

[18] Chen W, Ren X, He H, Zhou Y, Wu L (2020). Application of mapping allele with resolved carrier status technology in preimplantation genetic testing. Zhonghua Yi Xue Yi Chuan Xue Za Zhi.

[19] Shamash J, Rienstein S, Wolf-Reznik H, Pras E, Dekel M, Litmanovitch T, Brengauz M, Goldman B, Yonath H, Dor J, Levron J, Aviram-Goldring A (2011). Preimplantation genetic haplotyping a new application for diagnosis of translocation carrier's embryos-preliminary observations of two robertsonian translocation carrier families. J. Assist. Reprod. Genet..

[20] Suzuki T, Tsurusaki Y, Nakashima M, Miyake N, Saitsu H, Takeda S, Matsumoto N (2014). Precise detection of chromosomal translocation or inversion breakpoints by whole-genome sequencing. J. Hum. Genet..

[21] Obenauf AC, Schwarzbraun T, Auer M, Hoffmann EM, Waldispuehl-Geigl J, Ulz P, Günther B, Duba HC, Speicher MR, Geigl JB (2010). Mapping of balanced chromosome translocation breakpoints to the basepair level from microdissected chromosomes. J. Cell. Mol. Med..

[22] Treff NR, Thompson K, Rafizadeh M, Chow M, Morrison L, Tao X, Garnsey H, Reda CV, Metzgar TL, Neal S, Jalas C, Scott RT, Forman EJ (2016). SNP array-based analyses of unbalanced embryos as a reference to distinguish between balanced translocation carrier and normal blastocysts. J. Assist. Reprod. Genet..

[23] Baker M (2012). Structural variation: The genome's hidden architecture. Nat. Methods..

[24] Eid J, Fehr A, Gray J (2009). Real-time DNA sequencing from single polymerase molecules. Science.

[25] Clarke J, Wu HC, Jayasinghe L (2009). Continuous base identification for single-molecule nanopore DNA sequencing. Nat. Nanotechnol..

[26] Au CH, Ho DN, Ip BBK, Wan TSK, Ng MHL, Chiu EKW (2019). Rapid detection of chromosomal translocation and precise breakpoint characterization in acute myeloid leukemia by nanopore long-read sequencing. Cancer Genet..

[27] Chow JFC, Cheng HHY, Lau EYL, Yeung WSB, Ng EHY (2019). Highresolution mapping of reciprocal translocation breakpoints using long-read sequencing. MethodsX.

[28] Jeck WR, Lee J, Robinson H, Le LP, Iafrate AJ, Nardi V (2019). A nanopore sequencing-based assay for rapid detection of gene fusions. J. Mol. Diagn..

[29] Merker JD, Wenger AM, Sneddon T, Grove M, Zappala Z, Fresard L (2018). Long-read genome sequencing identifies causal structural variation in a Mendelian disease. Genet. Med..

[30] Van Assche E, Staessen C, Vegetti W, Bonduelle M, Vandervorst M, Van Steirteghem A, Liebaers I (1999). Preimplantation genetic diagnosis and sperm analysis by fluorescence in-situ hybridization for the most common reciprocal translocation t(11;22). Mol. Hum. Reprod..

[31] Yang Y, Liu Y, Ma P, Chen J, Ding T (2020). Application of next generation sequencing for preimplantation genetic test of 71 couples with one partner carrying a reciprocal or Robertsonian translocation. Zhonghua Yi Xue Yi Chuan Xue Za Zhi.

[32] De Gregori M, Ciccone R, Magini P, Pramparo T, Gimelli S, Messa J, Novara F, Vetro A, Rossi E, Maraschio P, Bonaglia MC, Anichini C, Ferrero GB, Silengo M, Fazzi E, Zatterale A, Fischetto R, Previderé C, Belli S, Turci A, Calabrese G, Bernardi F, Meneghelli E, Riegel M, Rocchi M, Guerneri S, Lalatta F, Zelante L, Romano C, Fichera M, Mattina T, Arrigo G, Zollino M, Giglio S, Lonardo F, Bonfante A, Ferlini A, Cifuentes F, Van Esch H, Backx L, Schinzel A, Vermeesch JR, Zuffardi O (2007). Cryptic deletions are a common finding in "balanced" reciprocal and complex chromosome rearrangements: A study of 59 patients. J. Med. Genet..

[33] Pollard MO, Gurdasani D, Mentzer AJ, Porter T, Sandhu MS (2018). Long reads: their purpose and place. Hum. Mol. Genet..

[34] Au CH, Ho DN, Ip BBK, Wan TSK, Ng MHL, Chiu EKW, Chan TL, Ma ESK (2019). Rapid detection of chromosomal translocation and precise breakpoint characterization in acute myeloid leukemia by nanopore long-read sequencing. Cancer Genet..

